# The mediating effect of life satisfaction between daily living abilities and depressive symptoms in the Chinese older people: evidence from CHARLS 2020

**DOI:** 10.3389/fpubh.2024.1393530

**Published:** 2024-08-15

**Authors:** Mingsheng Liao, Xuesi Zhang, Zhiquan Xie, Limei Li, Liqin Zou

**Affiliations:** ^1^Youth League Committee, Zhaoqing Medical College, Zhaoqing, Guangdong, China; ^2^Zhaoqing First People’s Hospital, Zhaoqing, Guangdong, China; ^3^School of Public Health, Zhaoqing Medical College, Zhaoqing, Guangdong, China

**Keywords:** older people, activities of daily living, disability, life satisfaction, depressive symptoms, mediating effect

## Abstract

**Background:**

Depressive symptoms represent a significant public health challenge, impacting the mental well-being of older adults. Despite this, the understanding of how activities of daily living (ADL) abilities correlate with life satisfaction and depressive symptoms among older Chinese adults remains limited.

**Aims:**

This study aims to investigate the relationship between ADL and depressive symptoms in older people Chinese individuals, with a specific focus on examining the mediating role of life satisfaction within this context.

**Methods:**

The study utilized data from the China Health and Retirement Longitudinal Study (CHARLS) collected in 2020. A cohort of 8,343 individuals aged 60 years and above was included. The analysis was conducted using STATA 17.0 and SPSS 26.0, employing descriptive statistics, chi-square tests, Pearson correlations, and mediation analysis using the percentile Bootstrap method with 5,000 resamples to explore the interrelations among ADL, life satisfaction, and depressive symptoms.

**Results:**

ADL is positively correlated with life satisfaction (r = 0.129, *p* < 0.01) and negatively correlated with depressive symptoms (r = −0.313, *p* < 0.01). Additionally, life satisfaction and depressive symptoms are negatively correlated with each other (r = −0.360, *p* < 0.01). In the model of the mediating effect, ADL directly, significantly and negatively predicts depressive symptoms in the Chinese older people (β = −0.193, t = −17.827, *p* < 0.001). After incorporating life satisfaction into the regression equation, the direct predictive effect of ADL on depressive symptoms remains significant (β = −0.177, t = −17.099, *p* < 0.001); furthermore, ADL has a significant positive predictive effect on life satisfaction (β = 0.007, t = 4.959, *p* < 0.001) and life satisfaction significantly negatively predicts depressive symptoms (β = −2.235, t = −27.799, *p* < 0.001). Furthermore, the direct effect of ADL on depressive symptoms (−0.177) and its mediating effect (−0.016) account for 91.71% and 8.29% of the total effect (−0.193), respectively.

**Conclusion:**

ADL is inversely associated with the risk of depressive symptoms among older people Chinese individuals, with life satisfaction serving as a significant mediator in this relationship. Interventions aimed at improving life satisfaction in older people individuals with ADL impairments may effectively reduce or prevent the onset of depressive symptoms.

## 1 Background

As global aging progresses, successful aging emerges as an effective strategy to address the challenges of population aging (1), drawing widespread attention. The concept of successful aging encompasses the absence of physical disabilities, the preservation of cognitive function, and mental well-being (2). Depression, a prevalent mental health issue, significantly impacts a vast number of older people individuals around the world. The prevalence of major depression, considered a leading cause of suicide among the older people, ranges from 5.37% to 56% (3). Depression in the older people frequently results in various adverse outcomes (4), such as deteriorations in physical health, diminished subjective well-being, and others (5). Therefore, understanding the mechanisms and pathways related to the onset of depression is crucial for achieving successful aging.

Activities of daily living abilities (ADL) refer to the ability to perform daily life tasks, which can be broadly divided into basic activities of daily living (BADL) and instrumental activities of daily living (IADL). ADL is crucial for maintaining independence and enhancing the quality of life in older adults. Previous research has demonstrated a significant positive correlation between ADL and depressive symptoms among the older people population (6, 7). For older people individuals residing alone, the impact of ADL on depression appears to be negligible, which has been reported by Lee and CHON (8) as well. Therefore, the impact of older people individuals’ ADL on depression has become convoluted.

Life satisfaction, a subjective measure of happiness, encapsulates an individual’s comprehensive evaluation of their life quality, predicated upon personal benchmarks. Based on the self-esteem theories (9), older people individuals with normal ADL abilities frequently display elevated levels of life satisfaction for the fulfilled self-esteem requirements (8). A study reveals that older people individuals without ADL disabilities reported approximately 20% higher levels of life satisfaction than those with ADL difficulty or ADL dependence (10). In addition, a large number of studies have shown that a negative relationship exists between life satisfaction and depression (11, 12) and a lower level of life satisfaction is a predictor of depression (5), whereas, another study has identified a positive association between the two variables (13). Consequently, the relationship between life satisfaction and depression among the older people has become unclear.

Overall, we hypothesize that while impairments in daily living abilities may directly lead to symptoms of depression, the extent of this impact may be moderated by the individual’s level of life satisfaction. The mediating role of life satisfaction in the relationship between daily living abilities and depression is an important yet underexplored aspect. The data from CHARLS 2020 (14), which includes a range of socioeconomic, health, and psychological variables among the older people population in China, provide an opportunity to empirically investigate this mediating effect. This study aims to contribute to a more comprehensive understanding of the factors affecting the psychological health and quality of life of the older people in China by exploring the mediating role of life satisfaction between daily living abilities and depression. These findings will offer reliable data support for the successful aging of the population.

## 2 Methods

### 2.1 Study sample

**Figure 1:**
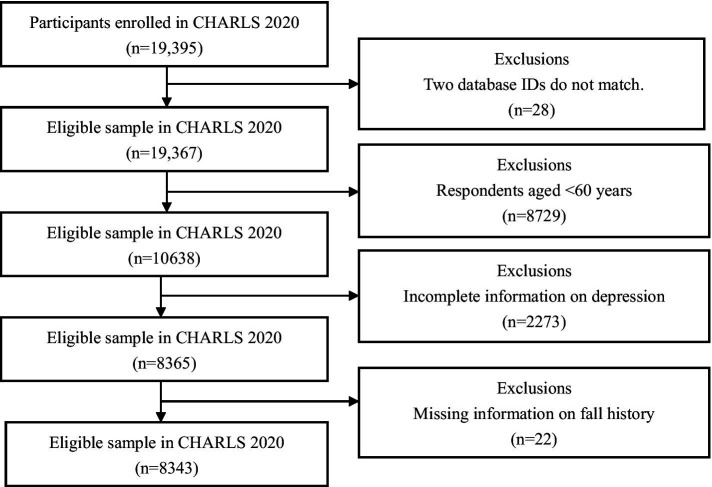
Flowchart of participant selection.

We used data from the 2020 CHARLS (China Health and Retirement Longitudinal Study), a nationally representative longitudinal survey of Chinese individuals aged ≥45 years and their spouses. This survey meticulously evaluates the social, economic, and health landscapes of community dwellers. Employing a sophisticated probability-proportional-to-size sampling methodology, the survey spanned 450 villages and 150 counties across 28 provinces (14). The study collected data through face-to-face interviews. Ethical approval was obtained from the Biomedical Ethics Review Committee of Peking University. The Peking University Public Data Management Agency agreed to our use of the data. We selected 8,343 respondents aged ≥ 60 years for the fixed-effects regression and mediation effect analysis (Figure 1).

### 2.2 Measures

#### 2.2.1 Measurement of depressive symptoms

In CHARLS, the CES-D-10 scale was used to estimate the risk of depressive symptoms. The CES-D-10 scale is composed of 10 items that assess the feelings of participants within the preceding week, with eight negative items and two framed positively. Scores for the negative items range from 0, indicating “rarely or none of the time,” to 3, representing “most or all of the time.” Conversely, the scoring for the positive items is inverted. Overall, the scale’s scores range from 0 to 30, where a higher score signifies greater severity of depressive symptoms and an increased likelihood of depression. Previous studies on the CES-D-10 scale suggested that the cutoff point for depressive symptoms in older adults is 10 (15, 16). Therefore, a cutoff score of 10 was set to distinguish the participants with or without depressive symptoms (score ≥ 10: high-risk depression; score < 10: low-risk depression). The CES-D-10 has shown good effectiveness and reliability in Chinese (17). In this study, Cronbach’s α was 0.788, with good reliability, and the value of KMO was 0.878, indicating good validity.

#### 2.2.2 Measurement of activities of daily living

The limitations of activities of daily living (ADL) were measured in terms of basic activities of daily living (BADL) and instrumental activities of daily living (IADL), which are based on the Katz Index of Independence in ADL (18) and the Lawton IADL Scale (19). This study incorporated six elements from BADL (toileting, eating, dressing, controlling urination and defecation, transferring in and out of bed, and bathing) alongside six components from IADL (making decisions regarding purchases and handling payments, telephone usage, meal preparation, conducting household chores, adhering to medication schedules with accurate dosages, and financial management). Scoring for each item was aligned with the criteria set by the Functional Independence Measure (FIM) scale (20, 21). Responses were categorized by calculating the average score in scenarios involving multiple levels of difficulty and scored as follows: “7 = do it without difficulty,” “6 = do it but with difficulty,” “4 = do it with difficulty and need help” and “1.5 = cannot do it” (22). In this study, the cumulative scores of the 12 items were then classified into two types of independence. The ADL total score was classified as Normal (i ≥ 72) indicating complete or conditional independence, and disability (i < 72) indicating conditional or complete dependence. The BADL total score, comprising the sum of the first six items, was classified as Normal (i ≥ 36). Similarly, the IADL total score, derived from the sum of the last six items, was classified as Normal (i ≥ 36). This scale displayed great internal consistency in the 2020 CHARLS sample, with Cronbach’s αat 0.853. Validity analysis showed that the KMO value was 0.913, with great validity.

#### 2.2.3 Assessment of life satisfaction

In this study, life satisfaction was assessed using item DC026 from the D Health Status and Function section of the “CHARLS” survey questionnaire. The question asked was, “Overall, are you satisfied with your life? Are you extremely satisfied, very satisfied, somewhat satisfied, not very satisfied, or not at all satisfied?” For the research, the response options were assigned values as follows: “5 = Extremely satisfied,” “4 = Very satisfied,” “3 = Somewhat satisfied,” “2 = Not very satisfied,” “1 = Not at all satisfied.” Thus, the higher the score, the greater the respondent’s level of life satisfaction.

#### 2.2.4 Control variables

The control variables in this study comprised gender, age (23), residence (24), marital status (25), self-rated health status (6, 26), history of falls (27, 28) (yes/no), and internet usage (29, 30) (yes/no). Gender was categorized into male and female groups. Age was segmented into three brackets: 60–69, 70–79, and 80 years and older. Residence classification included the main city zone, town, or others. Marital status was delineated as either cohabitating with a partner or living alone. Self-rated health status was assessed using the CHARLS data set scale, which ranks health on a scale from 5 (very good) to 1 (very poor).

### 2.3 Statistical analyses

The 2020 CHARLS data underwent a rigorous processing and filtering phase utilizing STATA 17.0, followed by a comprehensive analysis conducted with SPSS 26.0. This study employed descriptive statistical methodologies, including the calculation of means, standard deviations, frequencies, and percentages, to intricately delineate the socio-demographic attributes of the representative participants and the corresponding scores for each evaluated characteristic. To investigate the variations in ADL and depressive symptomatology across diverse socio-demographic groups, the chi-square test was meticulously applied. Furthermore, Pearson correlation coefficients were determined to elucidate the interrelations among ADL, life satisfaction, and depressive symptom scores. The mediating role of life satisfaction was examined through a percentile Bootstrap method incorporating bias correction, entailing 5,000 resamples, adhering to the ‘three-step method’ as advocated by Wen and Ye (31) (referenced in Figure 1). The statistical significance threshold for these tests was established at α = 0.05.

## 3 Results

### 3.1 Common method deviation test

To ensure statistical accuracy, this study employed Harman’s single-factor method and exploratory factor analysis. Eight components with eigenvalues over 1 were extracted, with the first factor explaining 20.40% of the variance, indicating no serious method bias (32).

### 3.2 Descriptive statistics and correlation analysis

As shown in Table 1, among the 8,343 older people Chinese participants, 41.04% reported depressive symptoms with an average score of 9.21. Most participants had normal ADL and BADL, at 93.58% and 96.45%, respectively, while 90.03% had normal IADL. The mean life satisfaction score was 3.30. The gender distribution was nearly balanced, with 4,186 males (50.17%) and 4,157 females (49.83%). The average age of participants was 67.96 years, ranging from 60 to 108 years, with a standard deviation of 6.02 years, and the majority were aged 60–69 years (66.06%). Additionally, 64.98% lived in towns or other areas, and 75.33% lived with a partner. Self-rated health varied, with 10.45% rating it as very good and 7.23% as very poor. A history of falls was reported by 18.37% of participants, and 24.69% used the internet.

**Table 1:** The descriptive statistics of the characteristics of the study population (*N* = 8,343).

Variables	*N*(%)	Mean(SD)
Depressive symptoms	3,424(41.04%)	9.21(6.56)
ADL		81.42(6.22)
Normal	7,841(93.58%)	
Disability	502(6.02%)	
BADL		41.18(2.41)
Normal	8,047(96.45%)	
Disability	296(3.55%)	
IADL		40.25(4.40)
Normal	7,511(90.03%)	
Disability	832(9.97%)	
Life satisfaction		3.30(7.76)
Gender		
Male	4,186(50.17%)	
Female	4,157(49.83%)	
Age		67.96(6.02)
60–69	5,511(66.06%)	
70–79	2,393(28.68%)	
≥80	439(5.26%)	
**Residence**		
Main city zone	2,922(35.02%)	
Town or other	5,421(64.98%)	
**Marital status**		
Living together with a partner	6,285(75.33%)	
Living alone	2,058(24.67%)	
**Self-rated health**		
Very good	872(10.45%)	
Good	956(11.46%)	
Fair	4,209(50.45%)	
Poor	1,703(20.41%)	
Very poor	603(7.23%)	
**History of falls**		
Yes	1,533(18.37%)	
No	6,810(81.63%)	
**Internet usage**		
Yes	2,060(24.69%)	
No	6,283(75.31%)	

Table 2 revealed significant socio-demographic differences in ADLs (ADL, BADL, and IADL) among the older people participants. Men exhibited higher rates of normal ADL (χ^2^ = 21.088), BADL (χ^2^ = 6.485), and IADL (χ^2^ = 50.048) than women. Younger participants (60–69 years) performed better in ADL (χ^2^ = 97.449), BADL (χ^2^ = 28.512), and IADL (χ^2^ = 136.700) compared to older age groups. Residence in main city zones was associated with better IADL outcomes (χ^2^ = 12.628). Married individuals had better IADL outcomes than those living alone (χ^2^ = 6.801). Self-rated health showed strong correlations, with the best health ratings corresponding to better ADL (χ^2^ = 718.880), BADL (χ^2^ = 391.905), and IADL (χ^2^ = 824.538) outcomes. Participants without a history of falls and those who used the internet also showed better outcomes, particularly in IADL (falls χ^2^ = 153.042, internet usage χ^2^ = 79.810).

**Table 2:** Social-demographic differences in ADL, BADL and IADL (*N* = 8,343).

Variables	ADL		χ^2^	BADL		χ^2^	IADL		χ^2^
	Normal	Disability		Normal	Disability		Normal	Disability	
Gender			21.088^***^			6.485^*^			50.048^***^
Male	3,984(95.17%)	202(4.83%)		4,059(96.97%)	127(3.03%)		3,871(92.47%)	315(7.53%)	
Female	3,857(92.78%)	300(7.22%)		3,988(95.93%)	169(4.07%)		3,640(87.56%)	517(12.44%)	
Age			97.449^***^			28.512^***^			136.700^***^
60–69	5,265(95.54%)	246(4.46%)		5,358(97.22%)	153(2.78%)		5,087(92.31%)	424(7.69%)	
70–79	2,201(91.98%)	192(8.02%)		2,274(95.03%)	119(4.97%)		2,086(87.17%)	307(12.83%)	
≥80	375(85.42%)	64(14.58%)		415(94.53%)	24(5.47%)		338(76.99%)	101(23.01%)	
Residence			2.634			3.312			12.628^***^
Main city zone	2,763(94.56%)	159(5.44%)		2,833(96.95%)	89(3.05%)		2,677(91.62%)	245(8.38%)	
Town or other	5,078(93.67%)	343(6.33%)		5,214(96.18%)	207(3.82%)		4,834(89.17%)	587(10.83%)	
Marital status			1.978			3.686			6.801^**^
Living with a partner	5,920(94.19%)	365(5.81%)		6,076(96.67%)	209(3.33%)		5,689(90.52%)	596(9.48%)	
Living alone	1,921(93.34%)	137(6.66%)		1,917(95.77%)	87(4.23%)		1,822(88.53%)	236(11.47%)	
Self-rated health			718.880^***^			391.905^***^			824.538^***^
Very good	863(98.97%)	9(1.03%)		869(99.66%)	3(0.34%)		648(97.36%)	23(2.64%)	
Good	939(98.22%)	17(1.78%)		946(98.95%)	10(1.05%)		912(95.40%)	44(4.60%)	
Fair	4,097(97.34%)	112(2.66%)		4,139(98.34%)	70(1.66%)		3,983(94.63%)	226(5.37%)	
Poor	1,501(88.14%)	202(10.86%)		1,582(92.89%)	121(7.11%)		1,390(81.62%)	313(18.38%)	
Very poor	441(73.13%)	162(26.87%)		511(84.74%)	92(15.26%)		377(62.52%)	226(37.48%)	
History of falls			173.365^***^			175.185^***^			153.042^***^
Yes	1,330(86.76%)	203(13.24%)		1,392(90.80%)	141(9.20%)		1,249(81.47%)	284(18.53%)	
No	6,511(95.61%)	299(4.39%)		6,655(97.72%)	155(2.28%)		6,262(91.95%)	548(8.05%)	
Internet usage			59.010^***^			27.324^***^			79.810^***^
Yes	2,008(97.48%)	52(2.52%)		2,025(98.30%)	35(1.70%)		1,960(95.15%)	100(4.85%)	
No	5,833(92.84%)	450(7.16%)		6,022(95.85%)	261(4.15%)		5,551(88.35%)	731(11.65%)	

^*^p < 0.05, ^**^p < 0.01, ^***^p < 0.001.

Table 3 indicated significant variations in depressive symptoms among participants based on gender (χ^2^ = 251.048, *p* < 0.001), age (χ^2^ = 8.273, *p* < 0.05), residence (χ^2^ = 153.089, *p* < 0.001), marital status (χ^2^ = 90.630, *p* < 0.001), self-rated health status (χ^2^ = 939.887, *p* < 0.001), history of falls (χ^2^ = 194.781, *p* < 0.001), and internet usage (χ^2^ = 159.162, *p* < 0.001).

**Table 3:** Social-demographic differences in Depressive symptoms (*N* = 8,343).

Variables	Depressive symptoms		χ^2^	*P*
	No depressive symptom	Depressive symptom		
Gender			251.048	0.000
Male	2,824(67.46%)	1,362(32.54%)		
Female	2,095(50.40%)	2,062(49.60%)		
Age			8.273	0.016
60–69	3,299(59.86%)	2,212(40.14%)		
70–79	1,353(56.54%)	1,040(43.46%)		
≥80	267(60.82%)	172(39.18%)		
Residence			153.089	0.000
Main city zone	1,988(68.04%)	934(31.96%)		
Town or other	2,931(54.07%)	2,490(45.93%)		
Marital status			90.630	0.000
Living together with a partner	3,890(61.89%)	2,395(38.11%)		
Living alone	1,029(50.00%)	1,029(50.00%)		
Self-rated health			939.887	0.000
Very good	718(82.34%)	154(17.66%)		
Good	756(79.08%)	200(20.92%)		
Fair	2,625(62.37%)	1,584(37.63%)		
Poor	672(39.46%)	1,031(60.54%)		
Very poor	148(24.54%)	455(75.46%)		
History of falls			194.781	0.000
Yes	661(43.12%)	872(56.88%)		
No	4,258(62.53%)	2,552(37.47%)		
Internet usage			159.162	0.000
Yes	1,459(70.83%)	601(29.17%)		
No	3,460(55.07%)	2,823(44.93%)		

### 3.3 Scores of ADLs, life satisfaction, and depressive symptoms and the correlations

As shown in Table 4, the participants exhibited moderate levels of depressive symptoms (mean 9.21, SD 6.56). The mean scores for ADL and life satisfaction were 81.42 (SD 6.22) and 2.70 (SD 0.78), respectively.

**Table 4:** Descriptive statistics and correlation analysis of ADL, Life satisfaction and Depressive symptoms.

	Score range	Mean	SD	1	2	3
1. ADL	18–84	81.42	6.22	1		
2. Life satisfaction	1–5	3.30	0.78	0.129^**^	1	
3. Depressive symptoms	0–30	9.21	6.56	−0.313^**^	−0.360^**^	1

^**^P < 0.01.

The results of the Pearson correlation test are presented in Table 4. ADL is positively correlated with life satisfaction (r = 0.129, *p* < 0.01) and negatively correlated with depressive symptoms (r = −0.313, *p* < 0.01). Additionally, life satisfaction and depressive symptoms are negatively correlated with each other (r = −0.360, *p* < 0.01).

Furthermore, Supplementary Tables 1, 2 demonstrated that both BADL and IADL had significant positive correlations with life satisfaction (r = 0.140 and r = 0.106, respectively, *p* < 0.01) and significant negative correlations with depressive symptoms (r = −0.284 and r = −0.288, respectively, *p* < 0.01).

### 3.4 Multicollinearity analysis among the independent variables

This study collected data through self-reported measures from participants, which may pose a risk of common method bias. Therefore using ADL and life satisfaction as independent variables, multiple linear regression analysis was conducted with the depressive symptoms of Chinese older people as the dependent variable. We assessed the variance inflation factor (VIF) to check for potential multicollinearity. The obtained VIF value of 1.017 is significantly lower than the threshold of 5 typically used to indicate substantial multicollinearity concerns. Similarly, the calculated tolerance level of 0.983 is considerably higher than the conventional minimum standard of 0.10. These results suggest that there are no multicollinearity issues in our model.

### 3.5 The results of regression and mediation analyses

Following the recommendations for testing the mediating effects by Wen and Ye (31), a regression analysis was conducted for the mediation model. The mediating effects were examined using a stepwise regression approach. The analysis results are presented in Table 3. In the regression and mediation analyses, the variables of gender, age, self-rated health, history of falls, and internet usage were all analyzed as covariates.

As shown in Table 5, ADL directly significantly and negatively predicts depressive symptoms in the Chinese older people (β = −0.194, t = −17.874, *p* < 0.001). After incorporating life satisfaction into the regression equation, the direct predictive effect of ADL on depressive symptoms remains significant (β = −0.178, t = −17.142, *p* < 0.001); furthermore, ADL has a significant positive predictive effect on life satisfaction (β = 0.007, t = 4.979, *p* < 0.001) and life satisfaction significantly negatively predicts depressive symptoms (β = −2.244, t = −27.821, *p* < 0.001).

**Table 5:** The regression analysis results of life satisfaction and depressive symptoms in the older people.

Variables	Model 1		Model 2		Model 3	
	β	t	β	t	β	t
Constant	16.430	15.219^***^	2.901	20.690^***^	22.941	21.665^***^
Gender	1.798	14.156^***^	0.021	1.250	1.844	15.177^***^
Age	−0.392	−3.559^***^	0.081	5.668^***^	−0.210	−1.989^*^
Self-rated health	1.960	29.845^***^	−0.184	−21.624^***^	1.546	23.947^***^
History of falls	−1.444	−8.654^***^	0.113	5.217^***^	−1.190	−7.444^***^
Internet usage	1.781	11.917^***^	0.020	1.032	1.825	12.771^***^
ADL	−0.194	−17.874^***^	0.007	4.979^***^	−0.178	−17.142^***^
Life satisfaction					−2.244	−27.821^***^
R^2^	0.236		0.079		0.301	
F	428.297^***^		119.622^***^		511.726^***^	

^*^P < 0.05; ^***^P < 0.001. Model 1,ADL predict depressive symptoms; Model 2, ADL predict life satisfaction; Model 3, ADL and life satisfaction jointly predict depression symptoms.

Similarly, the analysis was extended to BADL and IADL in Supplementary Tables 3, 4, respectively, showing consistent results: BADL significantly predicted depressive symptoms (β = −0.448, t = −16.179, *p* < 0.001), BADL significantly predicted life satisfaction (β = 0.023, t = 6.364, *p* < 0.001), BADL (β = −0.397, t = −14.942, *p* < 0.001) and life satisfaction (β = −2.235, t = −27.571, p < 0.001) significantly predicted depressive symptoms. IADL significantly predicted depressive symptoms (β = −0.246, t = −16.300, *p* < 0.001), IADL significantly predicted life satisfaction (β = 0.007, t = 3.486, *p* < 0.001), IADL (β = −0.230, t = −15.974, p < 0.001) and life satisfaction (β = −2.263, t = −28.383, *p* < 0.001) significantly predicted depressive symptoms.

**Figure 2:**
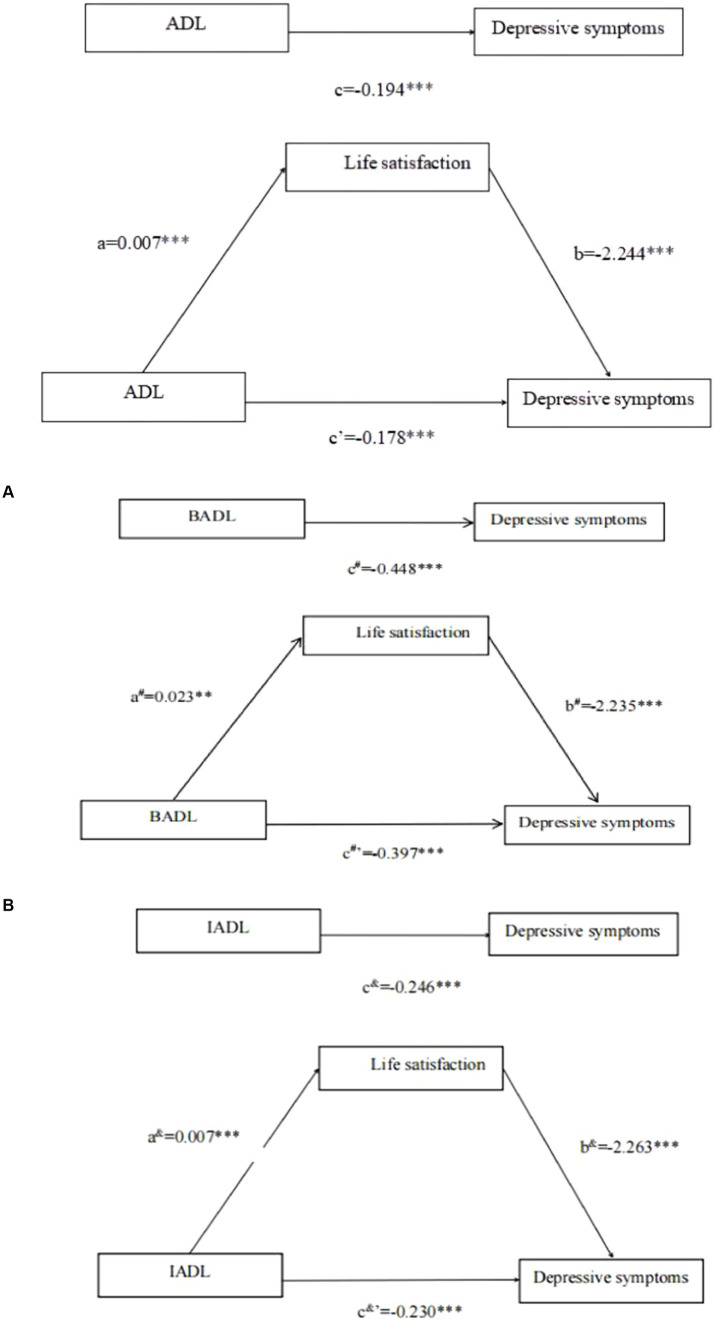
Total effect model and mediation model. ^a^Represents the effect value of ADL on Life satisfaction, ^b^represents the effect value of Life satisfaction on Depressive symptoms, ^c^represents the total effect value of ADL on Depressive symptoms, and ^c’^represents the direct effect value of ADL on depressive symptoms. ^***^p < 0.001. **(A)** ^a#^Represents the effect value of BADL on Life satisfaction, ^b#^represents the effect value on Life satisfaction on Depressive symptoms, c#represents the total effect value of BADL on Depressive symptoms, and c#’represents the direct effect value of BADL on Depressive symptoms. ^***^p < 0.001. **(B)** ^a&^Represents the effect value of IADL on Life satisfaction, ^b&^represents the effect value of Life satisfaction on Depressive symptoms, ^c&^represents the total effect value of IADL on Depressive symptoms, and ^c&’^represents the direct effect value of IADL on depressive symptoms. ^***^p < 0.001.

The deviation-corrected percentile bootstrap method (repeated sampling 5,000 times) was used to test the mediating effects of life satisfaction between ADLs (ADL, BADL, and IADL), and depressive symptoms. The results in Table 6 showed that the direct influence of ADL on depressive symptoms, coupled with its indirect influence mediated through life satisfaction, was assessed using bootstrap 95% confidence intervals, which were [−0.199, −0.158] for the direct effect and [−0.024, −0.008] for the indirect effect, respectively. Significantly, both intervals notably exclude 0, thereby confirming that ADL impacts depressive symptoms not merely in a direct manner but also indirectly through life satisfaction mediation. In detail, the direct influence of ADL on depressive symptoms (−0.178) and its mediated effect through life satisfaction (−0.016) contribute to 91.75% and 8.25% of the overall effect (−0.194), respectively. Figure 2 graphically delineates the mediating role of life satisfaction between ADL and depressive symptoms.

**Table 6:** Mediating effect of life satisfaction on ADL and depressive symptoms.

	Effect	SE	t	*P*	95%CI	
					LLCI	ULCI
Total effect	−0.194	0.011	−17.894	0.000	−0.215	−0.173
Direct effect	−0.178	0.010	−17.142	0.000	−0.199	−0.158
Indirect effect	−0.016	0.004^a^	-	-	−0.024^b^	−0.008^c^

CI, Confidence Interval; LLCI, Lower Limit Confidence Interval; SE, Standard Error; ULCL, Upper Limit Confidence Limit Interval. ^a^Bootstrap standard error. ^b^BootLLCI. ^c^BootULCL.

In Supplementary Tables 5, 6, the study found that both BADL and IADL significantly impact depressive symptoms. For BADL, the total effect (β = −0.448, *p* < 0.001) and the direct effect (β = −0.397, *p* < 0.001) were significant, with life satisfaction mediating this relationship (β = −0.051, *p* < 0.001). Similarly, for IADL, the total effect (β = −0.246, *p* < 0.001) and the direct effect (β = −0.230, *p* < 0.001) were significant, also with a significant mediation by life satisfaction (β = −0.016, *p* < 0.001). Figures 2A,B graphically illustrate the mediating role of life satisfaction between BADL, IADL, and depressive symptoms, respectively.

## 4 Discussion

Based on the above results, it was found that ADL was negatively associated with depressive symptoms, while positively associated with life satisfaction. Furthermore, life satisfaction showed a mediating effect between ADL and depressive symptoms.

In our study, 3,424 (41.04%) Chinese older people individuals exhibited depressive symptoms, a percentage higher than the 33.8% reported in CHARLS 2015 and lower than the 50.6% reported in CHARLS 2018 (6). To some extent, this indicated that the incidence of depressive symptoms has declined, but it remained significantly higher than the global average (3). In addition, ADL was significantly associated with depressive symptoms in Chinese older people individuals, which was consistent with previous research findings (6, 33). That may be attributed to the older people with superior daily living skills experiencing lower incidences of sleep disturbances, consequently leading to a reduced prevalence of depression (34, 35). In addition, a decline in daily living abilities is positively correlated with cognitive dysfunction in the older people (13). In contrast, older people individuals with cognitive dysfunction often had depressive symptoms (36). Therefore, individuals with impairments in daily living abilities are more susceptible to depression.

Life satisfaction is a crucial element for the model of successful aging (37, 38). Our results showed that ADL was positively associated with life satisfaction, which could be the result of high levels of social participation with normal IADL in the older people (39). Moreover, older people individuals with independence in daily living activities had a positive perception of their quality of life, which was related to psychological well-being (40). Furthermore, older people individuals with normal IADL experience lower caregiver burden, including reduced economic pressure and a more positive family atmosphere, thereby resulting in higher life satisfaction (41–43). In ADL-dependent/.

independent populations, satisfaction with family life, and economic status are significant components of high life satisfaction (10). In addition, there were some studies also confirmed that caregiver burden was negatively correlated with the older people’s BADL scores (44, 45). Therefore, it was inevitable that ADL was positively associated with life satisfaction.

Our research findings indicated that life satisfaction was negatively correlated with depression, which is consistent with the results of previous studies (46–48). That was to say, having a positive outlook on life and aging was crucial for preventing depression in the older people (48). Furthermore, one study found that life satisfaction negatively predicted depression (49), while, another survey of 300 patients with cardiovascular diseases revealed that 34.2% of the impact of life satisfaction on depression could be explained by self-esteem (50), which could be the main reason why older people individuals with normal ADL experience fewer instances of depression, primarily for their safeguarded self-esteem. However, there was a study discovered that life satisfaction was positively correlated with depression (13), which was contrary to the findings of this study. This might be due to differences in the occurrence of depression among older people individuals of different genders (23, 51). The study participants presenting divergent outcomes were exclusively older people males, in contrast to our investigation, which included a comprehensive cohort of older people individuals, with a nearly equal distribution between males and females. Consequently, the findings from our study offer a more representative understanding of the older people population as a whole, enhancing its generalizability to broader demographics.

Our findings showed that life satisfaction was a mediating factor between ADL and depressive symptoms among the older people cohort. Individuals with higher ADL were more likely to feel competent and in control of their lives, which could engender a sense of self-governance and independence (23) and then substantially augment their life satisfaction levels. Conversely, higher life satisfaction derived from better ADL ability could mitigate feelings of worthlessness or despair, reducing the risk or severity of depression (25). When older people individuals maintain or improve their ability to independently perform daily tasks, this positive self-assessment could boost their overall life satisfaction. Consequently, enhancing life satisfaction acted as a protective barrier against depression, cultivating a positive life perspective, resilience, and a renewed sense of purpose, which, in turn, diminished mental health vulnerabilities. older people individuals with greater independence were likely to engage more in social activities and sustain stronger social networks (52). Social support was acknowledged for its role in bolstering life satisfaction and safeguarding against depression through emotional support, diminution of isolation, and bolstered feelings of belonging (53, 54).

Hence, our study findings revealed that life satisfaction played a significant mediating role between ADL and depression. With aging, a decline in ADL is inevitable, while when older people individuals experienced ADL disabilities, interventions aimed at enhancing life satisfaction could reduce or prevent the onset of depression, thereby avoiding the adverse outcomes of depression.

## 5 Practical effect

This investigation carries profound practical ramifications for ameliorating the mental health of the Chinese older people. It elucidates the intermediary role of life satisfaction in bridging ADL and depression, shedding light on the potential of enhancing both the functional capabilities and overall life satisfaction of the older people to mitigate depressive symptoms. Additionally, this study accentuates life satisfaction’s mediating role, advocating that interventions designed to elevate life satisfaction may effectively shield against depressive manifestations stemming from ADL deficiencies. Practical applications of these findings could include the development of targeted programs that address both physical and psychological aspects of aging, such as physical therapy, social engagement activities, and psychological counseling, specifically designed to improve ADL capabilities and life satisfaction. This holistic approach could significantly decrease the prevalence of depressive symptoms in the older people, leading to better mental health outcomes and improved overall well-being in the aging population.

## 6 Strengths and limitations

This research utilized data from the China Health and Retirement Longitudinal Study (CHARLS), drawing on a sample that comprehensively represents the country’s demographic landscape.

The possible limitations of this study are: Firstly, due to the cross-sectional nature of the data used, causality cannot be established. While we found that life satisfaction mediates the relationship between ADL and depressive symptoms, the causal direction among these variables remains unclear. However, the China Health and Retirement Longitudinal Study (CHARLS) includes multiple waves of data, providing an opportunity for future research to conduct longitudinal analyses to better understand the temporal relationships among ADL, life satisfaction, and depressive symptoms. Secondly, although CHARLS data covers older people populations across China, the sample may not fully represent the diversity of all Chinese older people, particularly those residing in remote areas or specific sub-groups. Hence, the generalizability of the findings may be limited. Thirdly, the study considers a limited set of variables, the CHARLS dataset includes a rich array of variables related to health, economic status, and social factors. Future studies could leverage this comprehensive dataset to explore additional variables, such as social support and specific health conditions, to better understand the factors affecting ADL, life satisfaction, and depressive symptoms.

## 7 Conclusion

This study has illuminated a significant negative association between ADL and the prevalence of depressive symptoms among the older people in China, with life satisfaction playing a critical mediator. These findings advocate for the implementation of targeted interventions designed to improve life satisfaction as a strategic approach to prevent or reduce the onset of depression among older people individuals facing ADL impairments. Such interventions could potentially transform the mental health landscape for the older people, emphasizing the pivotal role of both physical and psychological well-being in combating depression.

## Data availability statement

Publicly available datasets were analyzed in this study. This data can be found at: https://charls.charlsdata.com/pages/data/111/zh-cn.html.

## Ethics statement

The studies involving humans were approved by the Biomedical Ethics Review Committee of Peking University. The studies were conducted in accordance with the local legislation and institutional requirements. The participants provided their written informed consent to participate in this study.

## Author contributions

ML: Conceptualization, Writing – original draft. XZ: Conceptualization, Writing – original draft. ZX: Methodology, Software, Writing – review & editing. LL: Formal analysis, Methodology, Writing – review & editing. LZ: Conceptualization, Funding acquisition, Writing – review & editing, Project administration.
