# Supplementary material for: The mediating effect of life satisfaction between daily living abilities and depressive symptoms in the Chinese older people: evidence from CHARLS 2020
Source: Front Public Health. 2024 Aug 15;12:1393530. doi: 10.3389/fpubh.2024.1393530 (PMC11357936; doi:10.3389/fpubh.2024.1393530)
Supplement: Supplementary file 1 [file Data_Sheet_1.doc]

# Supplementary Tables：

Table 1 Descriptive statistics and correlation analysis of BADL, Life satisfaction, and Depressive symptoms.

|  | Score range | Mean | SD | 1 | 2 | 3 |
| --- | --- | --- | --- | --- | --- | --- |
| 1 BADL | 9-42 | 41.18 | 2.41 | 1 |  |  |
| 2 Life satisfaction | 1-5 | 3.30 | 0.78 | 0.140** | 1 |  |
| 3 Depressive symptoms | 0-30 | 9.21 | 6.56 | -0.284** | -0.360** | 1 |

Note：

***P*<0.01

Table 2 Descriptive statistics and correlation analysis of IADL, Life satisfaction, and Depressive symptoms.

|  | Score range | Mean | SD | 1 | 2 | 3 |
| --- | --- | --- | --- | --- | --- | --- |
| 1 IADL | 9-42 | 40.25 | 4.40 | 1 |  |  |
| 2 Life satisfaction | 1-5 | 3.30 | 0.78 | 0.106** | 1 |  |
| 3 Depressive symptoms | 0-30 | 9.21 | 6.56 | -0.288** | -0.360** | 1 |

Note：

***P*<0.01

Table 3 The regression analysis results of life satisfaction and depressive symptoms in the elderly.

| Variables | Model 1a | | Model 2a | | Model 3a | | |
| --- | --- | --- | --- | --- | --- | --- | --- |
|  | β | t | β | t | | β | t |
| Constant | 18.391 | 14.384*** | 2.552 | 15.434*** | | 24.096 | 19.410*** |
| Gender | 1.875 | 14.728*** | 0.019 | 1.129 | | 1.916 | 15.724*** |
| Age | -0.270 | -2.454* | 0.078 | 5.513*** | | -0.094 | -0.897 |
| Self-rated health | 2.030 | 31.104*** | -0.183 | -21.707*** | | 1.620 | 25.231*** |
| History of falls | -1.410 | -8.396*** | 0.106 | 4.866*** | | -1.174 | -7.290*** |
| Internet usage | 1.846 | 12.330*** | 0.020 | 1.017 | | 1.890 | 13.186*** |
| BADL | -0.448 | -16.179*** | 0.023 | 6.364*** | | -0.397 | -14.942*** |
| Life Satisfaction |  |  |  |  | -2.235 | | -27.571*** |
| R^2^ | 0.231 | | 0.081 | | 0.295 | | |
| F | 416.176*** | | 122.456*** | | 497.801*** | | |

Note：

**P*<0.05; ****P*<0.001.

Model 1a: BADL predicts depressive symptoms; Model 2a: BADL predicts life satisfaction; Model 3a: BADL and life satisfaction jointly predict depression symptoms.

Table 4 The regression analysis results of life satisfaction and depressive symptoms in the elderly.

| Variables | Model 1b | | Model 2b | | Model 3b | | |
| --- | --- | --- | --- | --- | --- | --- | --- |
|  | β | t | β | t | | β | t |
| Constant | 7.447 | 8.517*** | 3.323 | 28.091*** | | 14.739 | 16.871*** |
| Gender | 1.680 | 13.161*** | 0.028 | 1.655 | | 1.743 | 14.294*** |
| Age | -0.535 | -4.812*** | 0.087 | 5.950*** | | -0.339 | -3.182** |
| Residence | 1.583 | 11.573*** | 0.020 | 1.137 | | 1.629 | 12.471*** |
| Marital status | 1.496 | 10.032*** | -0.050 | -2.548* | | 1.383 | 9.710*** |
| Self-rated health | 1.968 | 30.379*** | -0.189 | -22.261*** | | 1.540 | 24.182*** |
| History of falls | -0.514 | -9.195*** | 0.119 | 5.498*** | | -1.245 | -7.905*** |
| Internet usage | 1.256 | 8.122*** | 0.012 | 0.593 | | 1.283 | 8.689*** |
| IADL | -0.246 | -16.300*** | 0.007 | 3.486*** | | -0.230 | -15.974*** |
| Life Satisfaction |  |  |  |  | -2.263 | | -28.383*** |
| R^2^ | 0.251 | | 0.079 | | 0.317 | | |
| F | 348.582*** | | 89.045*** | | 429.272*** | | |

Note：

**P*<0.05;**P*<0.05; ****P*<0.001.

Model 1b: IADL predicts depressive symptoms; Model 2b: IADL predicts life satisfaction; Model 3b: IADL and life satisfaction jointly predict depression symptoms.

Table 5 Mediating Effect of Life satisfaction on BADL and Depressive symptoms.

|  | Effect | SE | t | *P* | 95%CI | |
| --- | --- | --- | --- | --- | --- | --- |
|  |  |  |  |  | LLCI | ULCI |
| Total effect | -0.448 | 0.028 | -16.179 | 0.000 | -0.502 | -0.393 |
| Direct effect | -0.397 | 0.027 | -14.942 | 0.000 | -0.449 | -0.345 |
| Indirect effect | -0.051 | 0.011^a^ | - | - | -0.072^b^ | -0.030^c^ |

Note:

CI: Confidence Interval.

LLCI: Lower Limit Confidence Interval.

SE: Standard Error.

ULCL: Upper Limit Confidence Limit Interval.

^a^ Bootstrap standard error.

^b^ BootLLCI.

^c^ BootULCL.

Table 6 Mediating Effect of Life satisfaction on IADL and Depressive symptoms.

|  | Effect | SE | t | *P* | 95%CI | |
| --- | --- | --- | --- | --- | --- | --- |
|  |  |  |  |  | LLCI | ULCI |
| Total effect | -0.246 | 0.015 | -16.300 | 0.000 | -0.275 | -0.216 |
| Direct effect | -0.230 | 0.014 | -15.974 | 0.000 | -0.258 | -0.202 |
| Indirect effect | -0.016 | 0.006^a^ | - | - | -0.027^b^ | -0.005^c^ |

Note:

CI: Confidence Interval.

LLCI: Lower Limit Confidence Interval.

SE: Standard Error.

ULCL: Upper Limit Confidence Limit Interval.

^a^ Bootstrap standard error.

^b^ BootLLCI.

^c^ BootULCL.
